# Langerhans Cell Histiocytosis of Maxilla and Mandible in 6 Years Old Child: A Case Report

**DOI:** 10.5005/jp-journals-10005-1029

**Published:** 2009-08-26

**Authors:** MK Jindal, Vivek Kumar Sharma, Ibyne Ahmed, Ashish Agrawal

**Affiliations:** 1 Chairman and Reader, Department of Pedodontics, Dr ZA Dental College, Aligarh Muslim University, Aligarh Uttar Pradesh, India; 2 Senior Lecturer, Department of Periodontics, Dr ZA Dental College, Aligarh Muslim University, Aligarh, Uttar Pradesh, India; 3 Professor, Department of Radiodiagnosis, JNMC, Aligarh Muslim University, Aligarh, Uttar Pradesh, India; 4 Postgraduate Student, Department of Periodontics, Dr ZA Dental College, Aligarh Muslim University, Aligarh Uttar Pradesh, India

## Abstract

Langerhans cell histiocytosis (LCH) is a group of idiopathic disorders characterized by the proliferation of specialized bone marrow-derived Langerhans cells (LCs) and mature eosinophils. Its etiology is unknown but it could be due to antigenic stimulus of an infectious, genetic abnormality, deregulated immune response, or even clonal origin. Clinical presentation may be localized and systemic, invading skin, lungs and bone in adult, and bone marrow and lymph node in children. Obtaining a biopsy that yields cells that are morphologically and immunohistochemically compatible with Langerhans cells, can make a definitive diagnosis of LCH. Poor prognosis factor include advanced age, disease extent and systemic organ abnormality. Conventional treatment of LCH is with surgery, radiotherapy, chemotherapy and steroid injections, alone or in combination. Spontaneous regression of localized disease has also been reported.

## INTRODUCTION

Langerhans cell histiocytosis (LCH) is a group of idiopathic disorders characterized by the proliferation of specialized bone marrow-derived Langerhans cells (LCs) and mature eosinophils.^1^ The nomenclature-histiocytosis X-was coined by Lichtenstein in 1953 to account for three clinical varieties which showed some histological characteristics in commoneosinophilic granuloma, Letterer-Siwe syndrome and Hand- Schuller-Christian syndrome. The term ‘histiocytosis’ refers to the proliferation of histiocytes and other inflammatory cells whereas the letter ‘X’ was added to denote the unknown etiology of the disease. The recent adoption of the terminology ‘Langerhans cell histiocytosis’ is due to the fact that the histiocytes involved in the disease present a phenotype which is similar to that of Langerhans cells found in normal mucosa and skin.^2^ A new proposed classification creates two categories of LCH- Nonmalignant disorders such as unifocal or multifocal eosinophilic granuloma and malignant disorders including Lettere siwe disease and variants of histiocytic lymphoma. The mandible is more frequently affected than the maxilla, with most of the lesions occurring in the molar area. Destruction of lamina dura results in the radiographic appearance of ‘floating teeth’. However, other bones may be affected such as the skull, long bones and ribs.^3^

The purpose of our study is to present a case history of a child patient with Langerhans cell histiocytosis with overt oral lesions.

## CASE REPORT

**Fig. 1:**
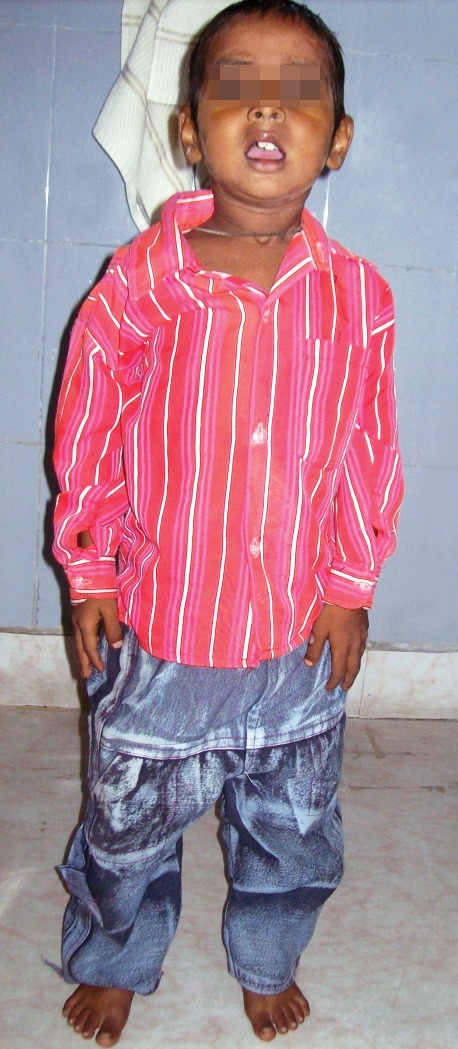
Standing view of child

A 6-year-old male child reported in the Department of Pedodontics, Dr ZA Dental college, AMU, Aligarh with the chief complaint of loosening of teeth, bleeding and swollen gums. The child had small stature (Fig. 1), cachetic, and mildly lethargic. All sensory and motor responses are with in normal limit.

Laboratory testing revealed total bilirubin of 0.5 mg/dl (normal 0.2-1.3), alkaline phosphatase 1277 (normal 250), SGOT 67 (normal 13), SGPT 46 (normal 14), a prothrombin time of 13 seconds (normal 12.6-15.2) and serum albumin level of 4.2 gm/dl (normal 3.5-4.8). Bone marrow iron was nil, suggest for iron deficiency anemia. Because the child appeared malnourished, a vitamin deficiency was suspected, but serum folate, vitamin A, vitamin B_12_, and vitamin D levels were within normal limits. The patient had a normal serum glucose level, and urine osmolality was markedly low at 4 mOsm/k (normal 300-900). Skin of scalp showed seborrheic dermatitis. All vital signs of the patient were in between normal limits. No abnormality in patient’s respiratory and blood vascular system were observed. Thyroid function test was normal; thyromegaly was seen but no feature of hypothyroidism. Hepatomegaly with no other organ dysfunction was too there.

Intraoral examination revealed poor oral hygiene, soft and erythmatous gingiva, bleeding on probing, multiple missing teeth, generalized tooth mobility and periodontal pocket with halitosis. Submandibular lymphadenopathy was present.

**Fig. 2:**
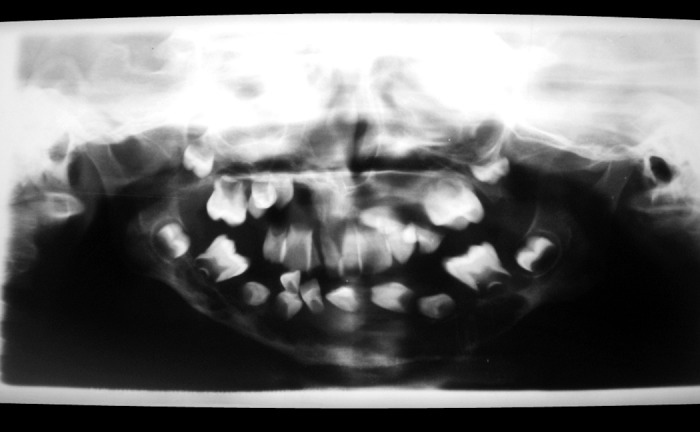
OPG view

**Fig. 3:**
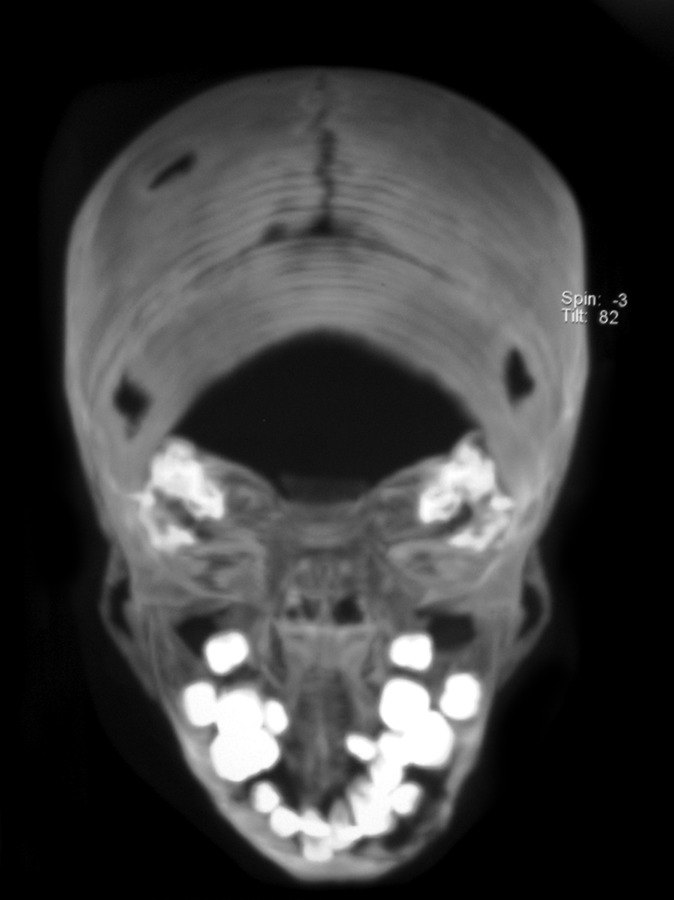
CT-view of skull

**Fig. 4:**
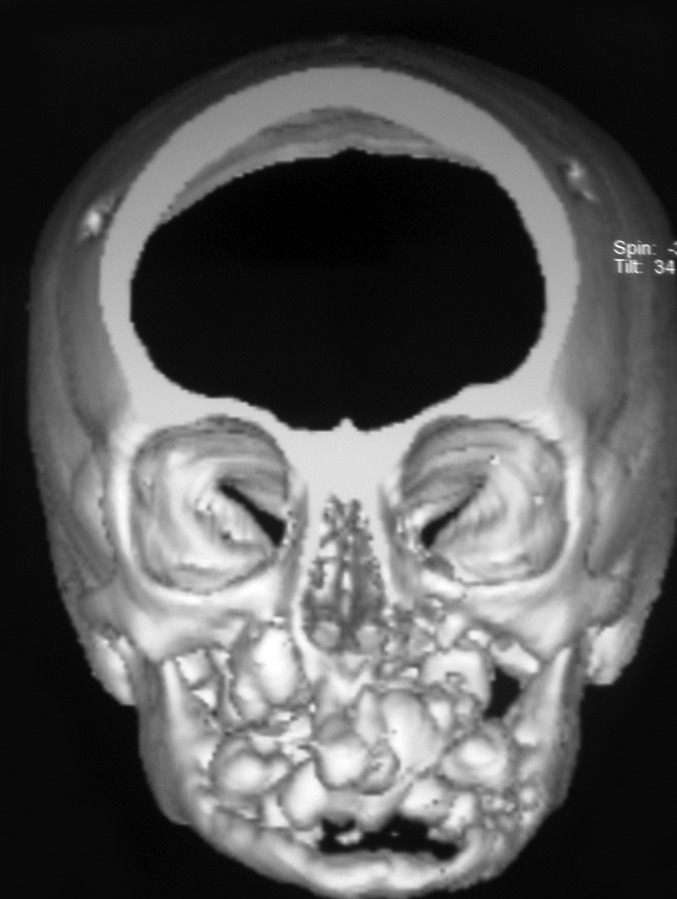
3D constructive view of CT-head

**Fig. 5:**
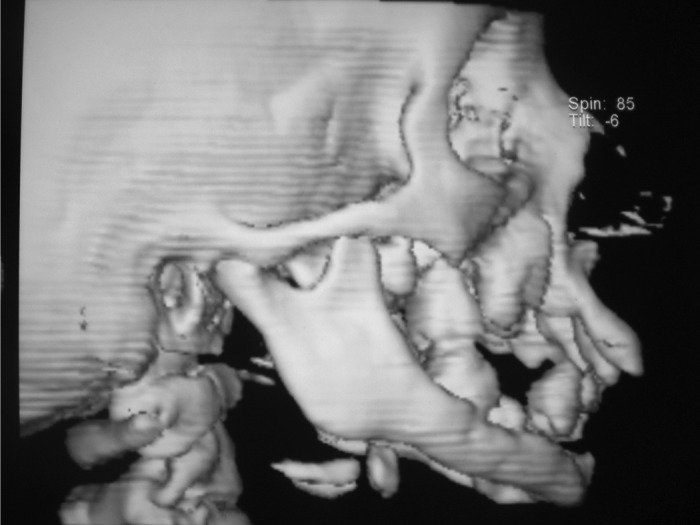
3D reconstructive view right side of CT-face

**Fig. 6:**
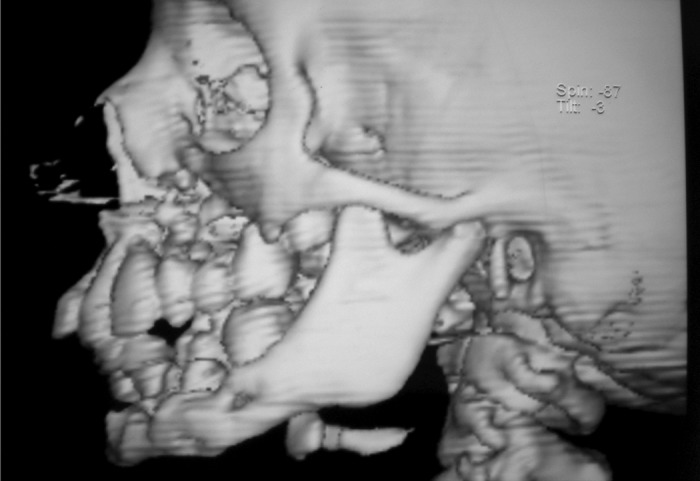
3D reconstructive view left side of CT-face

**Fig. 7:**
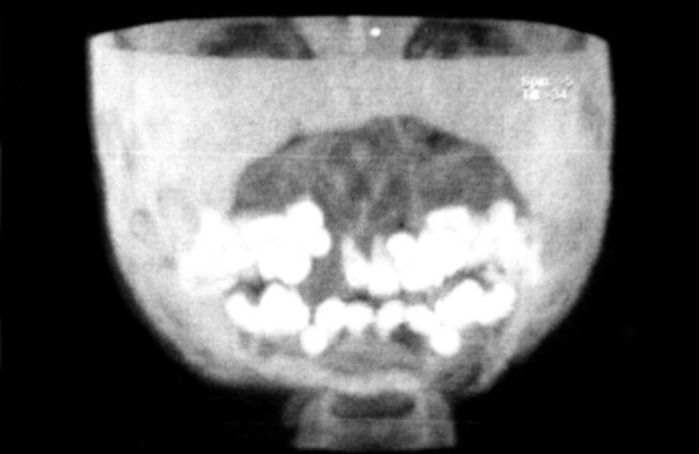
CT-image showing floating teeth

**Fig. 8:**
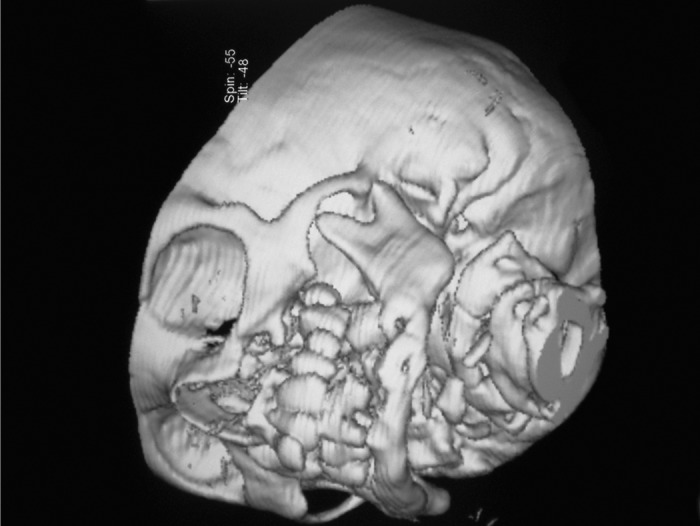
3D view of base of skull CT-image

**Figs 9A and B:**
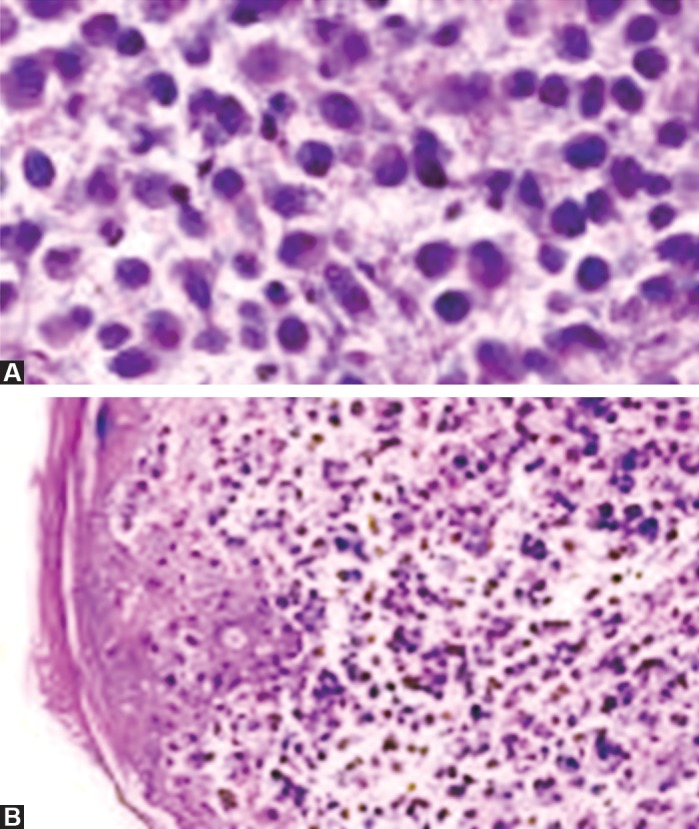
Biopsy of mandible

For radiographic examination panoramic view was taken. This showed multiple radiolucent lesions in maxilla and mandible with generalize alveolar bone loss, providing the radiographic image of ‘floating teeth’ (Fig. 2). Computed tomography of head shows multiple sharply marginated radiolucent lesions involving the calvaria, mainly parital bone associated with small extra-axial soft tissue component (Figs 3 to 8). Tomography of face revealed lytic lesion involving alveolus of mandible associated with minimal soft tissue component with tooth appearing to be floating inside with lytic lesion involving alveolus of maxilla and mandible. All these features were suggestive of a case of histiocytosis. Skin biopsy of the patient showed Langerhans’ cell histiocytosis with marked CD1a and S-100. Mandible biopsy showed larged number of histiocytes (CD-68 and S-100 positive), which is too suggestive for Langerhans cell histiocytosis. Electron microscopy showed the presence of Birbeck granules (Figs 9A and B). Bone marrow biopsy showed large number of histiocytes (CD-68 and S-100 positive) mixed with neutrophlic and lymphocytes. Overall it was a clear case of Langerhans cell histiocytosis involving both mandible and maxilla along with skull too.

## DISCUSSION

Histiocytosis is a term applied to a group of rare disorders of the reticuloendothelial system, the disorder in the category of LCH are abnormality resulting from proliferation of specialized bone marrow derived antigen presenting dendritic cells (Langerhans cells) and mature eosinophills. Langerhans cell histiocytosis may involve almost any organ system, but the frequency of involvement, as well as the extent of the disease, is often age dependent.^4^ The incidence of LCH ranges from 0.5 to 5.4 cases per million persons per year, depending upon the age of the population investigated. Although the disease can present at any age, it usually presents within the first few years of life and has a slight male predominance.^5^

Eosinophlic granuloma usually appears in the skeleton and in rare case in soft tissues, head and neck lesion are common at initial presentation of LCH. Lesion may cause dull and steady pain, in jaws the disease may cause bony swelling, soft tissue mass, pain, gingivitis, ulceration and loosening of teeth often occur after the destruction of alveolar bone.^6^ The disseminated form may involve multiple bony lesions, diabetes insipidus and exaphthalmos, the condition know as Hand-Schuller-Christian disease. Letter Siwe disease most often occurs in the infant under 3 year of age. In this soft tissue and granulomatous reactions disseminated throughout the body and the condition is marked by intermittent fever, hepatosplenomegaly, anemia, lymphadenopathy and failure to thrive.

The criteria for diagnosis of LCH includes identification of the characteristic clinical features, but also requires histopathological and immunohistochemical findings of the involve lesions.^7^ Since 50-80 percent of cases manifest cutaneous involvement, a skin biopsy provides a rapid and accessible means to secure the diagnosis. A definitive diagnosis of LCH requires that the lesional cells exhibit positive staining with S-100 and CD1a.^8^ The histiocyte society has established a set of guidelines to assist in the diagnosis and study of LCH, these guidelines consists of a complete physical examination, height and weight measurements, hematological assays and coagulation studies, liver function tests, and urine osmolality. Bone marrow examination is required when symptoms or blood tests suggest its involvement. For definitive diagnosis a complete skeletal radiographic survey and chest radiography is also required. Patients with identified abnormalities require more specific studies, such as pulmonary function tests and lung biopsy, small bowel series, liver biopsy, panoramic view of jaws, computed tomography of head and faces, endocrine evaluation and otolaryngoscopy. Since patients with LCH often have chronic and recurrent disease, follow-up studies are required every month to 6 months, depending upon organ system involvement and the degree of organ dysfunction.^7^ The case reported here presented signs similar to those of severe periodontal disease, namely gingival bleeding, severe tooth mobility, halitosis and alveolar bone loss, both in the maxilla and in the mandible. Along with biopsy report the computed tomography of head and face of our 6-year male child patient was similar to the criteria for histiocyte society.

Conventional treatment of LCH is with surgery, radiotherapy, chemotherapy and steroid injections, alone or in combination as indicated by the extent of the disease.^9^ Spontaneous regression of localized disease has also been reported.^10^

Consequently, all patients with LCH require long-term follow-up to identify disease recurrence or late-stage complications. Lastly, clinicians should be cognizant that patients with LCH are at risk for second malignancies, including solid tumors and hematopoietic conditions.^11^

## CONCLUSION

Langerhans cell histiocytosis is a rare disease, the etiology and pathogenesis of which remain unknown. A variety of etiological factors have been proposed including immunologic reactions, viruses, bacteria and genetic influence. Numerous reports stress the fact that oral manifestations may be among the earliest signs of the disease, which cause patients to seek treatment. Since most of children visit pedodontist first with chief complaint of multiple mobile teeth, carefull clinical examination and good diagnostic skill let the earlier referral and treatment of patient to specialist concern, thereby increasing survival chances with minimal deformity. A wide spectrum of treatment modalities has been adopted to deal with Langerhans' cell histiocytosis, including wide surgical excision together with radiotherapy. Other treatments have been suggested such as chemotherapy, isolated radiotherapy and the use of alkalizing agents.

## References

[1] Watanabe K (1990). Prepubertal periodontitis: A review of diagnostic
criteria, pathogenesis, and differential diagnosis. J Periodont Res.

[2] Rapidis AD, Langdon JD, Harvey PW, Patel  MF (1978). Histiocytosis
X. An analysis of 50 cases. Int J Oral Surg.

[3] Hartman KS (1980). Histiocytosis X: a review of 114 cases with oral
involvement. Oral Surg Oral Med Oral Pathol.

[4] Longaker MA, Frieden IJ, LeBoit PE, Sherertz EF (1994). Congenital
"self-healing" Langerhans cell histiocytosis: The need for longterm follow-up. J Am Acad Dermatol.

[5] Alston RD, Tatevossian RG, McNally RJ, Kelsey A, Birch JM, Eden TO (2007). Incidence and survival of childhood Langerhans cell
histiocytosis in Northwest England from 1954 to 1998. Pediatr
Blood Cancer.

[6] (2004). Predictors of outcome in children with Langerhans cell
Histiocytosis. ASG, Annual meeting proceedings (postmeeting
edition). J Clin Oncol.

[7] Broadbent V, Gadner H, Komp DM, Ladisch S (1989). Histiocytosis
syndromes in children: II. Approach to the clinical and laboratory
evaluation of children with Langerhans cell histiocytosis. Clinical
Writing Group of the Histiocyte Society. Med Pediatr Oncol.

[8] Favara BE, Feller AC, Pauli  M, Jaffe EC, Weiss  LM, Arico M, Bucsky  P, Egeler RM, Elinder  G, Gadner H (19997). Contemporary
classification of histiocytic disorders. The WHO Committee On
Histiocytic/Reticulum Cell Proliferations. Reclassification
Working Group of the Histiocyte Society. Med Pediatr Oncol.

[9] Roychoudhury A, Shah N, Parkash H, Mukhopadhyay S, Chopra P (1998). Eosinophilic granuloma of the jaws. Br J Oral
Maxillofac Surg.

[10] Broadbent V, Pritchard J, Davies EG, Levinsky  RJ, Heaf D, Atherton  DJ,  Pincott JR,  Tucker S (1984). Spontaneous remission of
multi-system histiocytosis X. Lancet.

[11] Longaker MA, Frieden IJ, LeBoit PE, Sherertz  EF (1994). Congenital
"self-healing" Langerhans cell histiocytosis: the need for longterm
follow-up.. J Am Acad Dermatol.

